# Dental anxiety, psychological distress and oral health behavior in 263 patients from Albania and Germany

**DOI:** 10.1186/s13104-026-07681-1

**Published:** 2026-01-31

**Authors:** Nertsa Cunoti, Rezart Qorri, Erda Qorri, Lisa Irmscher, Hendrik Berth

**Affiliations:** 1 Carl Gustav Carus Faculty of Medicine, Division of Psychological and Social Medicine and Developmental Neurosciences, Research Group Applied Medical Psychology and Medical Sociology, TUD Dresden University of Technology, Fetscherstr. 74, 01307 Dresden, Germany; 2 Department of Dentistry, Faculty of Medical Sciences, Albanian University, Tirana, 1001 Albania

**Keywords:** Dental anxiety, Dental fear, Depression, Somatization, Anxiety, Psychological distress, Albania, Germany, Oral health, Dental care

## Abstract

**Objectives:**

This dataset provides information on dental anxiety and psychological distress experienced by patients in Albania and Germany. Data were collected using the Dental Anxiety Scale (DAS) and the Brief Symptom Inventory-18 (BSI-18). Additional information on oral health and dental care behaviors was also obtained.

**Data description:**

The dataset contains the responses from 263 patients (*N* = 133 German and *N* = 130 Albanian) who completed questionnaires between December 2019 and July 2020. The variables include measures of dental anxiety (DAS), psychological distress (BSI-18), and self-reported oral health behaviors such as the tooth brushing frequency, frequency of dental visits, frequency of tartar removal, and frequency of professional teeth cleaning. The dataset also includes measures of subjective health status and oral health-related self-efficacy. The dataset enables for cross-cultural comparisons and supports further research into the relationship between dental anxiety, psychological distress, and oral health practices.

**Supplementary Information:**

The online version contains supplementary material available at 10.1186/s13104-026-07681-1.

## Objective

Despite continuous progress in medical science, dental anxiety remains a widespread condition. Studies show that nearly 80% of adults in industrialised countries feel anxious before dental treatment, 20% have a genuine fear of procedures, and around 5% avoid dental care entirely [1, 2]. This phenomenon is present across all age groups, with children also exhibiting avoidance behaviours, that are often influenced by parental attitudes [3].

Several standardized tools have been developed to measure dental anxiety. The Dental Anxiety Scale (DAS), introduced by Corah in 1969, remains one of the most widely used instruments [4, 5]. It consists of four questions focusing on situations before and during dental treatment. The validity and reliability of the DAS have been demonstrated in numerous studies [6]. Another widely used tool is the Brief Symptom Inventory-18 (BSI-18) by Derogatis [7]. The BSI-18 measures psychological distress with only 18 items, making it a concise yet effective tool [8]. There are three scales – depression, anxiety and somatisation – each consisting of six items that contribute to the Global Severity Index (GSI), providing a total score for psychological distress.

In this study, patients completed a set of questionnaires assessing their oral health behaviours. The questions covered how often they brushed their teeth, how regularly they visited the dentist, how often they had tartar removal and professional cleanings, and how confident they were in their ability to maintain good oral health.

The study aims to improve understanding of the psychological and behavioral dimensions linked to dental treatment by collecting and analysing these data. Furthermore, the dataset is expected to be valuable for researchers in Germany, Albania, and beyond, as it will allow them to compare the differences in results across countries and cultural contexts [9]. The dataset could also be used for secondary analyses, such as cross-cultural validation and psychometric testing.

## Data description

This dataset (data file 1 in Table 1) [10] provides responses from 263 patients who completed the Dental Anxiety Scale (DAS) [5], the Brief Symptom Inventory-18 (BSI-18) [8], and a set of descriptive questions on oral health and preventive behavior prior to receiving dental treatment. Data were collected between December 2019 and July 2020 from two locations: a dental clinic in Plauen, Germany (*n* = 133) and a dental clinic in Tirana, Albania (*n* = 130). Patients between the ages of 14 and 80 participated voluntarily after providing written informed consent (for the *n* = 3 minors, consent was obtained from parents or legal guardians). Patients were included if they had sufficient knowledge of German (for patients in Germany) or Albanian (for patients in Albania), could complete the questionnaire, and had no evident psychiatric symptoms. The questionnaires were administered by dentists in the waiting room before treatment. At the time of the study no validated Albanian versions of the BSI-18 or DAS existed. Therefore, for Albanian patients, the validated German versions were translated into Albanian, so that each study participant could complete the questionnaire in their native language. The translation and back-translation were conducted by legally accredited bilingual translators from the Faculty of Foreign Languages in Tirana, Albania, to ensure both linguistic and conceptual fidelity. The study team (dentists: NC, RQ, EQ, psychologists: LI, HB) discussed the translations to ensure that the content and language of both versions are consistent. Several team members speak both Albanian and German (NC, RQ). Due to the scope of the study, a formal evaluation of the measurement equivalence of the German and Albanian versions of BSI-18 and DAS could not be carried out. The Albanian version of the questionnaire is provided as supplementary material.

None of the participants required assistance with completing the forms. The absence of psychiatric symptoms was assessed by the study staff. No detailed symptom assessment was performed, such as the use of a psychiatric checklist. However, the study staff paid attention to symptoms such as temporal and spatial disorientation, disturbances in speech flow, speech tempo and volume, addictive behaviour (e.g. the smell of alcohol), gait disturbances, delusions, hallucinations, severe physical agitation, irritability and outbursts of anger, severe depression, compulsive behaviour, impaired concentration and memory, loss of reality, confusion and severe neglect of personal appearance. None of the potential participants showed any psychiatric symptoms and therefore had to be excluded from participating in the study. This rules out any selection bias. The dataset includes information on country of residence, sex, age, health status, DAS scores (items 1–4) and BSI-18 scores (items 1–18), as well as responses to oral health behavior questions (items 1–5).

**Table 1:** Overview of data files/data sets

Label	Name of data file/data set	File types (file extension)	Data repository and identifier (DOI or accession number)
Data file 1	Dental_Anxiety_Albania_Germany.xlsx	MS Excel File (.xlsx)	OpARA— Research Data Repository of Saxon Universities 10.25532/OPARA-273 [10]
Data file 2	Data description_Dental_Anxiety_Albania_Germany.pdf	Portable Document File (.pdf)	OpARA—Research Data Repository of Saxon Universities 10.25532/OPARA-273 [10]

The mean age of all participants was 38 years, with a higher proportion of females in Germany (67.7%) and Albania (52.3%) [9].

Health status was measured using the question “How would you rate your current state of health?”. Responses were scored from 1 to 5, 1 indicate “very good”, 2 indicate “good”, 3 indicate “satisfactory”, 4 indicate “less good” and 5 indicate “poor”. The Dental Anxiety Scale (DAS) [8] comprises four questions each scored from 1 to 5, where “1” indicates no discomfort while “5” represents extreme fear. Questions 1 and 2 assess situations before treatment, while questions 3 and 4 focus on experiences during treatment. Dental anxiety is calculated as the sum of the four items. The possible range of values on the scale is between 4 and 20. Higher values indicate greater dental anxiety. The Brief Symptom Inventory-18 (BSI-18) comprises 18 items divided into three subscales: Somatization (items 1, 4, 7, 10, 13 and 16), Depression (items 2, 5, 8, 11, 14 and 17), and Anxiety (items 3, 6, 9, 12, 15 and 18). The Global Severity Index (GSI) total score was calculated by summing up all 18 items. Participants were asked to rate the extent to which they had experienced the described feeling over the past six days, on a scale from 0 (“not at all”), 1 “a little bit”, 2 (“moderately”), 3 (“quite a bit”) to 4 (“extremely”). The possible range of values for the three subscales is between 0 and 30, and between 0 and 90 for the total GSI score. Higher values indicate greater somatization, depression, anxiety or psychological distress.

Participants were also asked to report on their oral health behaviors using a similar scaled format. Toothbrushing frequency was recorded as follows 0 (never), 1 (once daily), 2 (twice daily), 3 (three times daily), 4 (four times daily) or 5 (more often). The frequency of dental visits, tartar removal and professional teeth cleaning was recorded as follows 0 (never), 1 (once a year), 2 (twice a year), 3 (three times a year), 4 (four times a year) or 5 (more often). Participants also rated their perceived ability to maintain oral health (self-efficacy) on a five-point scale ranging from 1 (“nothing at all”), 2 (“little”), 3 (“some”), 4 (“much”) to 5 (“very much”).

Missing data may have arisen if participants forgot individual items or deliberately voided answering individual items. These are coded as ‘999.00’ in the dataset and are therefore easily identifiable. The entire dataset (263 participants, 36 variables, 8,496 fields) contains *N* = 19 missing values (0.22%). There are no missing values in the overall scores for DAS and BSI-18. No participants had to be excluded due to having too many missing values.

Descriptive statistics were used to check all variables in the data set for anomalies or inconsistencies. For example, individual DAS items must be between 1 and 5 (not 6 or any other value) and the total score must be 4 or greater but not greater than 20.

Data file 2 [10], see Table 1] is a help file that describes each field belonging to data file 1. This description is also included as a separate sheet in the data file 1.

### Limitations

The sample size is relatively small with only 130–133 people surveyed in each country. Conducting the study in only two dental clinics limits how generalisable the results are. All patients completed the questionnaires themselves. For patients under 18 years of age parental consent was obtained. Some patients may not have answered truthfully, providing minimal responses to avoid being identified as anxious. Dental anxiety and psychological distress were assessed using short questionnaires. While efficient, these brief screening tools may not capture complex constructs sufficiently, potentially introducing measurement bias. As no validated Albanian versions of the questionnaires were available, legally accredited bilingual translators translated the German versions for this study. Despite following this rigorous translation procedure, no formal psychometric validation of the Albanian versions has yet been conducted, which may limit cross-cultural comparisons.

Additionally, socioeconomic variables such as income level, employment status, and insurance coverage could influence understanding of dental anxiety. These variables were not included in the data collection. Objective indicators of oral health, such as the Periodontal Screening Index (PSI) or the DMF-T index, were also unavailable. The data were collected in 2019/2020, but processing and uploading to the data repository were delayed due to the Coronavirus pandemic outbreak. The dataset may underrepresent individuals with severe dental anxiety, as such patients often avoid dental treatment altogether. In addition, the expected treatment following completion of the survey was not recorded. Patients experiencing acute pain may have been more psychologically vulnerable and more anxious than those awaiting routine dental procedures.

## Supplementary Information

Below is the link to the electronic supplementary material.

Supplementary Material 1.

## Data Availability

The data described in this Data note can be freely and openly accessed on OpARA - Research Data Repository of Saxon Universities under under http://dx.doi.org/10.25532/OPARA-273. Please see Table 1 and references [10] for details and links to the data.

## Abbreviations

BSI-18: Brief Symptom Inventory-18
DAS: Dental Anxiety Scale
DMT-T: Decayed, Missing, Filled Teeth
GSI: Global Severity Index, total Score of the Brief Symptom Inventory-18
PSI: Periodontal Screening Index
