# Supplementary material for: Dental anxiety, psychological distress and oral health behavior in 263 patients from Albania and Germany
Source: BMC Res Notes. 2026 Jan 31;19:94. doi: 10.1186/s13104-026-07681-1 (PMC12947531; doi:10.1186/s13104-026-07681-1)
Supplement: Supplementary file 1 — Supplementary Material 1. [file 13104_2026_7681_MOESM1_ESM.pdf]

TECHNISCHE UNIVERSITÄT DRESDEN

Projekt kerkimor: Faktoret ndikues tek pacientet anksioze ne kabinetin dentar– Nje krahasim ndermjet Shqiperise dhe Gjermanise

Fakulteti i Mjekesise Carl Gustav Carus, Grupi kerkimor aplikues i mjekesise ne Psikologji dhe Sociologji
Albanian University, Tirane

Kodi:_____

1. Gjinia ❐ femer ❐ mashkull **2.** Mosha: ______ vjet **3.** Nr. i femijeve: ______

4. Gjendja Civile ❐ beqar/e ❐ i/e martuar ❐ lidhje afatgjate ❐ i/e ndare ❐ i/e ve

5. Bashkejetoni? ❐ po ❐ jo

6. Arsimimi ❐ Fillore ❐ 8-9 vjecare ❐ Gjimnaz ❐ Shkolle Profesionale ❐ asnje

7. Arsimi i larte ❐ Universitet ❐ asnje

8. Aftesia per te punuar: ❐ po ❐ jo ❐ ne pension ❐ shtepijak/e ❐ pa pune ❐ tjeter

9. Vendi i Punes: ❐ punetor/e ❐ i/e punesuar ❐ zyrtare ❐ privat ❐ te tjera

10. Kur keni qene per here te fundit tek dentisti? (Muaji/Viti) ______/______ ❐ Nuk shkoj asnjehere tek dentisti.

11. Cili eshte qellimi i vizites se sotme tek dentisti?
❐ Kontroll ❐ Pastrim profesional ❐ Probleme me mishrat e dhembeve
❐ Trajtim i planifikuar ❐ Dhimbje ❐ te tjera

12. Si do ta pershkruanit gjendjen tuaj aktuale te gojes? ❐ shume mire ❐ mire ❐ kenaqshem ❐ keq ❐ shume keq

13. Paramendoni se neser do te shkoni tek dentisti: Si ndjeheni? *Ju lutem kryqezoni vetem nje pegjijge!*

❐ Une shkoj me kenaqesi tek dentisti.
❐ Me duhet pak guxim te shkoj tek dentisti.
❐ Nuk e kam problem.
❐ Une e parandiej qe do te jete e dhimbshme dhe e sikleteshme.
❐ Une kam shume frike dhe shqetesohem se cfare do te beje dentisti me mua.

**Mendoni sikur,**	**i/e qete**	**pak e pa-kendshme**	**i/e tensio-nuar**	**i/e frike-suar**	**kaq i/e frikesuar sa qe me dalin djerse te ftohta dhe ndjehem si i semure.**
**1.** Ndodheni ne dhomen e pritjes tek dentisti. Si ndjeheni?	❐	❐	❐	❐	❐
**2.** Ndodheni tek poltroni dentar dhe dentisti pergatit instrumentat per te punuar tek dhembi juaj. Si ndjeheni?	❐	❐	❐	❐	❐
**3.** Ndodheni tek poltroni dentar per te pastruar dhembet. Nderkohe qe prisni, dentisti pergatit instrumentat per te hequr gurezat. Si ndjeheni?	❐	❐	❐	❐	❐

Ju lutem vini re faqen mbrapa!

Ju lutem kryqezoni theniet e meposhtme mbi trajtimin dentar te planifikuar.	aspak				shume
1. Jam shume i shqetesuar per trajtimin dentar.	❒ 1	❒ 2	❒ 3	❒ 4	❒ 5
2. Trajtimi dentar me vjen here pas here ne mendje.	❒ 1	❒ 2	❒ 3	❒ 4	❒ 5

Këtu është një listë e problemeve dhe ankesave që ndonjëherë i keni. Ju lutemi rishikoni çdo pyetje me kujdes dhe vendosni sa jeni shqetësuar ose ngacmuar nga këto ankesa **gjatë 7 ditëve të fundit** deri më sot. Ju lutemi bëni vetëm një kryq pas secilës pyetje me përgjigjen që ju përshtatet më së miri.

		aspak	shume pak	pak	e forte	shume e forta
01.	Zbehje dhe marramendje	❒	❒	❒	❒	❒
02.	Ndjesia, nuk me intereson asgje	❒	❒	❒	❒	❒
03.	Nervozitet dhe dridhje te brendshme	❒	❒	❒	❒	❒
04.	Dhimbje zemre dhe gjoksi	❒	❒	❒	❒	❒
05.	Ndjenja e vetmise	❒	❒	❒	❒	❒
06.	Ndjeheni te tensionuar	❒	❒	❒	❒	❒
07.	Ju vjen per te vjelle ose shqetesime ne stomak	❒	❒	❒	❒	❒
08.	Ndjeheni melankolik	❒	❒	❒	❒	❒
09.	Frike e papritur dhe pa arsye	❒	❒	❒	❒	❒
10.	Veshtiresi ne frymemarrje	❒	❒	❒	❒	❒
11.	Ndjeheni pa vlere	❒	❒	❒	❒	❒
12.	Sulme te frikshme ose paniku	❒	❒	❒	❒	❒
13.	Ndjesi mpirjeje ose shpim gjilperash ne trup	❒	❒	❒	❒	❒
14.	Ndjehesh i pa shprese perballe se ardhmes	❒	❒	❒	❒	❒
15	Humbja e qetesise	❒	❒	❒	❒	❒
16.	Ndjenja e dobesise ne trup	❒	❒	❒	❒	❒
17.	Mendime per te kryer vetevrasje	❒	❒	❒	❒	❒
18.	Ndjesi tmerri	❒	❒	❒	❒	❒

	kurre	1 x	2 x	3 x	4 x	shpesh
1. Sa here ne dite i lani dhembet?	❒	❒	❒	❒	❒	❒
2. Sa here ne vit shkoni tek dentisti?	❒	❒	❒	❒	❒	❒
3. Sa here ne vit pastroni vetem gurzat e dhembeve tek dentisti?	❒	❒	❒	❒	❒	❒
4. Sa here ne vit beni pastrim profesional dhembesh tek dentisti?	❒	❒	❒	❒	❒	❒
5. Nese mendoni per dhembet tuaj, si eshte gjendja e tyre?	❒ shume keq	❒ keq	❒ kenaqshem	❒ mire	❒ shume mire	

6. A keni anulluar apo humbur nje takim dentar si pasoje e frikes ndaj dentistit?
❒ po ❒ jo

7. Sa mund te beni vete per te ruajtur ose permiresuar shendetin e dhembeve tuaj?
❒ absolutisht shume ❒ shume ❒ disi ❒ pak ❒aspak

8. A keni frike para nje trajtimi dentar?
❒ absolutisht shume frike ❒ shume frike ❒ pak frike ❒ aspak frike ❒ absolutisht aspak frike

Ju lutem vini re faqen mbrapa!

	kurre	pak	pak a shume	shpesh	shume shpesh
**1.** A keni patur veshtiresi ne pertypjen e ushqimeve, per shkak te problemeve me dhembet ose/dhe protezat tuaja gjate muajit te kaluar?	❒	❒	❒	❒	❒
**2.** Gjate muajit te kaluar keni patur dhimbje ne hapesiren e gojes?	❒	❒	❒	❒	❒
**3.** A jeni ndjere te pakendshem gjate muajti te kaluar per shkak te paraqitjes se dhembeve ose/dhe protezave tuaja?	❒	❒	❒	❒	❒
**4.** A keni patur pershtypjen qe gjate muajit te kaluar, per shkak te dhembeve ose/dhe protezave tuaja, ushqimet nuk ju kane shijuar?	❒	❒	❒	❒	❒
**6.** A keni patur veshtiresi gjate muaji te kaluar ne ndjekjen e aktivi teteve te perditeshme per shkak te problemeve me dhembet dhe/ ose protezat tuaja?	❒	❒	❒	❒	❒

1. Tek sa njerez mund te mbeshteteni nese keni probleme serioze personale? Do thoja...
1❒ asnje 2❒ 1 deri 2 3❒ 3 deri 5 4❒ me shume se 5

2. Sa interes dhe simpati tregojne njerzit e tjere ne ate qe ju beni?
1❒ absolutisht shume 2❒ shume 3❒ as shume as pak 4❒ pak 5❒ asnje interes dhe simpati

3. Sa e lehte eshte per ju te merrni ndihme nga fqinjet kur keni nevoje per ta? Eshte...
1❒ shume e thjeshte 2❒ e thjeshte 3❒ e mundshme 4❒ e veshtire 5❒ shume e veshtire

Ju lutemi klasifikoni reagimet tuaja kur beni nje trajtim dentar.

	aspak	pak	disi	shume	absolutisht shume
**1.** Muskujt e mi tensionohen	❒	❒	❒	❒	❒
**2.** Frymemarrja me pershpejtohet	❒	❒	❒	❒	❒
**3.** Une djersij	❒	❒	❒	❒	❒
**4.** Me perzihet dhe me dhemb stomaku	❒	❒	❒	❒	❒
**5.** Me pershpejtohen rrahjet e zemres	❒	❒	❒	❒	❒

Optimistet jane njerez qe shikojne me besim te ardhmen e tyre dhe kryesisht presin gjera te mira. Ju lutemi vleresoni veten: Sa optimist jeni ju ne pergjithesi?	**aspak optimist**	❒	❒	❒	❒	❒	❒	❒	**shume optimisti**
Pesimistet jane njerez qe me dyshim shikojne te ardhmen e tyre dhe zakonisht presin gjera te keqija. Ju lutemi vleresoni veten: Sa pesimist jeni ju ne pergjithesi?	**aspak pesimist**	❒	❒	❒	❒	❒	❒	❒	**shume pesimist**

Tani behet fjale per kenaqesine tuaj te pergjthshme te jetes. Sa i kenaqur jeni sot me jeten tuaj?

aspak I kenaqur **shume i kenaqur**

❒	❒	❒	❒	❒	❒	❒	❒	❒	❒	❒
0	1	2	3	4	5	6	7	8	9	10

Shume faleminderit per bashkepunimin!
